# The role of physical therapy in the treatment of lipedema: an integrative review of therapeutic strategies and the current clinical landscape

**DOI:** 10.1590/1677-5449.202501962

**Published:** 2026-07-21

**Authors:** Débora Aparecida Oliveira Modena, Jaqueline Munaretto Timm Baiocchi, Elaine Caldeira de Oliveira Guirro

**Affiliations:** 1 Universidade de São Paulo – USP, Faculdade de Medicina de Ribeirão Preto – FMRP, Departamento de Ciências da Saúde, Ribeirão Preto, SP, Brasil.; 2 Centro Universitário de Jaguariúna – UniFAJ, Jaguariúna, SP, Brasil.; 3 Instituto Lipedema Brasil, São Paulo, SP, Brasil.

**Keywords:** lipedema, physical therapy modalities, conservative treatment, lipedema, modalidades de fisioterapia, tratamento conservador

## Abstract

Lipedema is a chronic disorder characterized by a disproportionate accumulation of subcutaneous adipose tissue in the extremities, typically sparing the hands and feet. It is commonly associated with pain, hypersensitivity, easy bruising, edema, and fibrotic nodules. Despite growing awareness, there is still no consensus on evidence-based therapeutic approaches. This integrative review aimed to identify physical therapy strategies described in the scientific literature for the assessment and conservative management of lipedema. Searches were conducted in the PubMed/MEDLINE, BVS, PEDro, Embase, and Cochrane databases, followed by critical appraisal and evidence synthesis, with applicable PRISMA-ScR checklist items used as a reporting guide for the study selection process. The findings indicate that physical therapy plays a key role in conservative treatment, particularly through complete decongestive therapy, compression therapy, exercise, manual therapy, and electrophysical agents. These interventions show potential benefits in reducing pain, improving functional capacity, controlling edema, and preventing disease progression. However, the available evidence remains limited, underscoring the need for more robust clinical trials to support evidence-based physical therapy practice in the treatment of lipedema.

## Resumo

O lipedema é uma doença crônica caracterizada pelo acúmulo desproporcional de tecido adiposo subcutâneo nos membros, poupando as mãos e os pés. É comumente associado a dor, hipersensibilidade, facilidade para hematomas, edema e nódulos fibróticos. Apesar da crescente conscientização, ainda não há consenso sobre abordagens terapêuticas baseadas em evidências. Esta revisão integrativa teve como objetivo identificar estratégias fisioterapêuticas descritas na literatura científica para a avaliação e o tratamento conservador do lipedema. Foram realizadas buscas nas bases de dados PubMed/MEDLINE, BVS, PEDro, Embase e Cochrane, seguidas de avaliação crítica e síntese das evidências, com uso de itens aplicáveis do checklist PRISMA-ScR como guia para o relato do processo de seleção dos estudos. Os resultados indicam que a fisioterapia desempenha um papel fundamental no tratamento conservador, particularmente por meio de terapia descongestiva complexa, terapia compressiva, exercícios, técnicas manuais e agentes eletrofísicos. Essas intervenções demonstram benefícios potenciais na redução da dor, melhora da função, controle do edema e prevenção da progressão da doença; no entanto, as evidências disponíveis ainda são limitadas, o que ressalta a necessidade de ensaios clínicos mais robustos para embasar a prática da fisioterapia baseada em evidências.

## INTRODUCTION

Lipedema is a chronic, progressive, and multifactorial condition that predominantly affects women. It is characterized by an abnormal and symmetrical accumulation of subcutaneous adipose tissue, primarily in the lower extremities, hips, and arms, with little or no involvement of the hands and feet. This accumulation is typically resistant to volume reduction, even following interventions such as dietary modifications, physical exercise, and, in some cases, traditional bariatric surgery, making both diagnosis and treatment challenging.^1-3^

Although initially described by Irving Phillips Lyon in 1910 and later detailed by Allen and Hines in 1940, lipedema was only officially recognized as a distinct disorder of subcutaneous fat in 2022, with its inclusion in the 11th Revision of the International Classification of Diseases (ICD-11) under codes EF02.2 and BD93.1Y.^4,5^ The estimated prevalence of lipedema ranges from 0.06% to 11% in the general female population, with higher rates reported in countries such as Germany (39%) and Brazil (12.3%). Recent population-based data suggest that approximately 8.8 million Brazilian women aged 18 to 69 years may have symptoms suggestive of lipedema.^2-5^

The etiology of lipedema remains uncertain, although its multifactorial nature is recognized. Genetic predisposition with an autosomal dominant inheritance pattern has been frequently reported, with a positive family history identified in up to 60% of cases.^6-8^ Hormonal changes, particularly estrogen-related dysfunctions in adipocytes (such as hypertrophy and hyperplasia), and microvascular alterations involving lymphatic and blood capillaries have also been proposed as pathophysiological mechanisms.^6-9^

From a clinical standpoint, the most common symptoms are spontaneous pain or pain to light touch, a feeling of heaviness, edema that worsens throughout the day (especially during standing), and a tendency to bruise even in the absence of overt trauma. Additional manifestations include subcutaneous nodules, joint hypermobility, muscle weakness, and biomechanical changes such as lower extremity misalignment, foot arch abnormalities, and impaired mobility. In more advanced stages, patients may develop skin lesions, tissue maceration, recurrent infections, varicosities, and secondary lymphedema (also known as lipo-lymphedema).^9-11^

In addition to its physical manifestations, lipedema can substantially impair functional capacity and quality of life, interfering with daily activities, autonomy, and social relationships. Emotional distress is also frequently reported, including eating disorders, reduced self-esteem, and depressive symptoms.^2-5^

The treatment of lipedema requires a multidisciplinary approach aimed at alleviating symptoms, slowing disease progression, and managing potential complications. Although there is still no cure, several therapeutic strategies have been proposed. Conservative treatment typically includes dietary interventions, regular physical exercise, compression therapy, and physical therapy. The latter is considered fundamental for pain relief, edema reduction, and improvement of physical function.^5,8,10,11^

Despite the variety of physical therapy interventions described in the literature, the relative effectiveness and safety of these approaches remain unclear. In this context, this integrative review aimed to explore the main physical therapy strategies used in the management of lipedema, encompassing assessment, diagnosis, and therapeutic interventions. Additionally, we sought to discuss the physiological effects associated with these interventions and evaluate their effectiveness in controlling pain, edema, and lymphatic function, thus contributing to a better clinical and scientific understanding of the role of physical therapy in the comprehensive care of individuals with lipedema.

## METHODS

### Guiding question

This integrative review was conducted to answer the following research question: What physical therapy strategies have been described in the scientific literature for the assessment and treatment of lipedema?

### Study design and reporting framework

This study was designed as an integrative review, which allows the synthesis of evidence derived from different study designs to support clinical practice in physical therapy. To enhance the transparency and reproducibility of reporting, particularly regarding study identification, screening, and eligibility assessment, applicable items from the Preferred Reporting Items for Systematic Reviews and Meta-Analyses Extension for Scoping Reviews (PRISMA-ScR) checklist were used as a reporting guide. The use of PRISMA-ScR was limited to reporting purposes and did not alter the integrative nature of the review.

### Information sources and search strategy

A comprehensive and systematic literature search was conducted to ensure representativeness and minimize selection bias. Searches were performed between March and August 2025 in the following international and regional electronic databases: PubMed/MEDLINE, Biblioteca Virtual em Saúde (BVS), PEDro, Embase, and the Cochrane Central Register of Controlled Trials (CENTRAL). In addition, the reference lists of all included studies were manually screened to identify potentially relevant publications not retrieved through the electronic search.

The search strategy combined controlled vocabulary terms, including Medical Subject Headings (MeSH) and Health Sciences Descriptors (DeCS), and free-text terms related to lipedema and physical therapy interventions. The initial search strategy was developed and conducted in PubMed, considering its specific indexing structure, and was subsequently adapted to the other databases by adjusting syntax, Boolean operators, and search fields while maintaining the original conceptual framework.

To ensure reproducibility, the complete PubMed search strategy is as follows: ("Lipedema" [MeSH] OR lipedema OR lipoedema OR lipo-lymphedema) AND ("Physical Therapy Modalities" [MeSH] OR physiotherapy OR "physical therapy" OR "manual therapy" OR "lymphatic drainage" OR exercise OR compression OR "electrophysical agents").

### Inclusion and exclusion criteria

Studies were considered eligible if they were original investigations or review articles addressing physical therapy strategies for lipedema, ranging from functional assessment to conservative therapeutic interventions. No restrictions were imposed regarding language, year of publication, or sample size. Studies were excluded if they were duplicate records, if their primary focus was not related to physical therapy approaches, or if they lacked clinically relevant data (such as editorials, letters to the editor, and commentaries).

### Study selection

Study selection was conducted in 3 sequential steps: title screening, abstract reading, and full-text review of potentially eligible studies. The process was performed independently by 2 reviewers (DAOM and JMTB), with verification by a third reviewer (ECOG) in case of disagreement.

### Critical appraisal and level of evidence

The included studies were critically appraised to assess methodological rigor and practical applicability. The strength of evidence was classified according to the hierarchy proposed by the Oxford Centre for Evidence-Based Medicine^12^ and adapted by Souza et al.^13^ for use in integrative reviews. The levels of evidence were defined as follows:

- Level 1: meta-analyses of randomized controlled trials (high evidence)
- Level 2: individual randomized controlled trials (high to moderate evidence)
- Level 3: quasi-experimental studies (moderate evidence)
- Level 4: non-experimental or descriptive studies (low to moderate evidence)
- Level 5: case reports or case series (low evidence)
- Level 6: expert opinions or consensus (very low evidence, although clinically relevant)

Only original clinical studies were classified according to this hierarchy. Review articles, consensus statements, and conceptual papers were included to provide contextual support but were not assigned a level of evidence. The appraisal process was conducted independently by 2 reviewers (DAOM and JMTB), and disagreements were resolved by consensus with a third reviewer (ECOG).

## RESULTS

**Figure 1:**
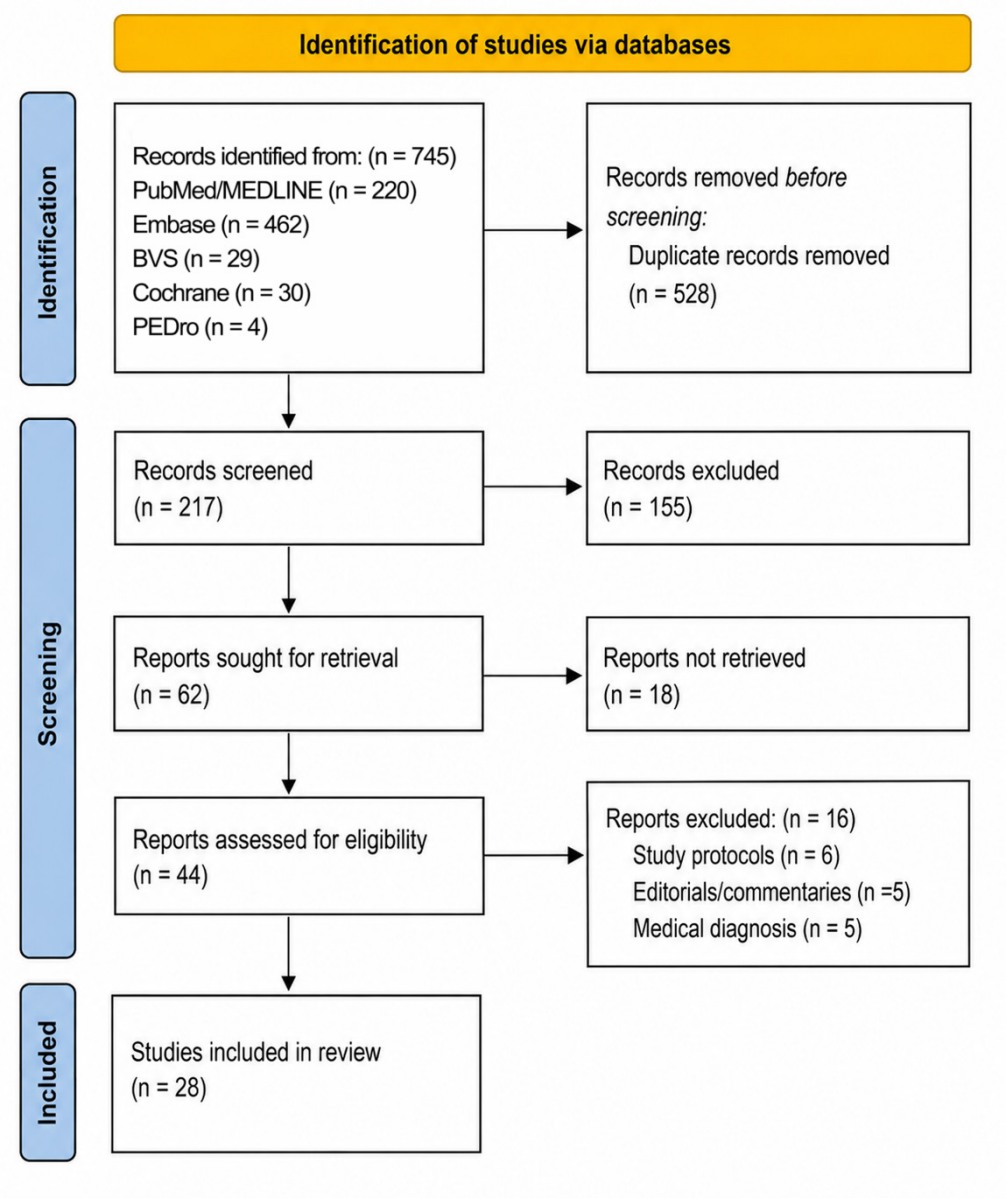
Flowchart of the study selection process.

The database search identified 745 records. After removal of duplicates, 217 records remained for title and abstract screening. Of these, 155 studies were excluded, and 44 full-text articles were assessed for eligibility. Following full-text review, 16 studies were excluded for not meeting the inclusion criteria, resulting in the inclusion of 28 studies in this integrative review. The study selection process is illustrated in Figure 1.

The included studies presented different methodological designs, with a predominance of observational, descriptive, and quasi-experimental studies. Few randomized controlled trials were available to date. The main characteristics and findings of the included studies are summarized in Table 1, including authors, year of publication, study design, sample, methods or interventions investigated, main outcomes, and level of evidence.

**Table 1:** Summary of evidence from studies included in the integrative review.

**Summary of evidence from studies included in the integrative review**					
***Physical therapy assessment and diagnosis of lipedema***					
**Reference**	**Study design**	**Sample**	**Method / intervention**	**Main outcomes**	**Level of evidence**
Amato et al.**^14^**	Validation study	154 women with lipedema	QuASiL questionnaire translated, culturally adapted, and validated in Brazil.	Validated a practical and comprehensible (96.4%) Brazilian Portuguese version of the QuASiL.	Level 4 – low to moderate
Bauer et al.**^10^**	Descriptive study	209 women with stage 2 lipedema after tumescent liposuction	Online questionnaire, assessing symptoms and comorbidities.	Liposuction reduced pain, swelling, and bruising and improved QoL.	Level 4 – low to moderate
Czerwińska et al.**^15^**	Scoping review	10 studies	Various tools: circumference, volume, US, VAS, SF-36, QuASiL, LEFS, 6MWT.	Identified and summarized tools used to assess outcomes of conservative lipedema treatment.	Level 4 – moderate
Kruppa et al.**^16^**	Narrative review / expert consensus	Literature-based	Clinical assessment guidelines: history, inspection, palpation, and differential diagnosis.	Summarized pathophysiology hypotheses and provided diagnostic and treatment recommendations; highlighted absence of biomarkers and limited evidence for therapies.	Level 5-6 – low
***Complete decongestive therapy***					
Szolnoky et al.**^17^**	Quasi-experimental study	21 women with lipedema	MLD, pneumatic compression, and bandaging.	Significantly reduced limb volume and decreased capillary fragility.	Level 3 – moderate
Atan and Bahar-Özdemir**^18^**	RCT	33 women with severe lipedema	3 groups: CDT + exercise, pneumatic compression + exercise, and exercise alone.	All groups improved, but CDT + exercise was superior for limb volume, pain, and physical function.	Level 2 – high
Donahue et al.**^19^**	Interventional pilot study	5 women with early-stage lipedema	9 multimodal physical therapy sessions (CDT, exercise, compression, education) + sodium MRI.	Pain reduction (VAS), functional improvement (PSFS), QoL improvement (RAND-36), and tissue sodium reduction (inflammation marker).	Level 3 – moderate
Schmeller and Meier-Vollrath**^21^**	Consensus / guideline	—	Clinical recommendations.	Suggested CDT as initial approach in advanced lipedema.	Level 6 – consensus
Faerber et al.**^20^**	German S2k guideline	—	Normative document.	Reinforced CDT as a central conservative management strategy.	Level 6 – guideline/consensus
Herbst et al.**^22^**	U.S. consensus guideline	—	Clinical guideline.	Highlighted CDT benefits for drainage, edema control, pain relief, and tissue integrity.	Level 6 – consensus
***Compression therapy***					
Czerwińska et al.**^23^**	Interventional pilot study	6 women with lipedema	Compression leggings + exercise program.	Compression + exercise reduced pain and bruising, maintained or decreased limb circumference, and improved QoL.	Level 3 – moderate
Paling and Macintyre**^24^**	Cross-sectional survey	279 women with lipedema	Structured online questionnaire on symptoms and use of compression garments.	70% used compression garments; main benefits: support (73%), pain reduction (67%), and mobility improvement (54%); but satisfaction was low due to discomfort and donning difficulty.	Level 4 – low to moderate
Ricolfi et al.**^25^**	Observational pilot study	29 women with type III lipedema	Micromassage compression leggings + moderate physical activity.	Improved spontaneous and evoked pain, reduced limb volume and subcutaneous adipose tissue thickness, and improved skin texture.	Level 3 – moderate
***Motor and musculoskeletal physical therapy***					
van Esch-Smeenge et al.**^26^**	Comparative cross-sectional study	44 women (22 with lipedema, 22 with obesity)	Muscle strength (MicroFET™) and 6MWT.	Women with lipedema showed significantly lower quadriceps strength and a trend toward reduced functional capacity.	Level 3 – moderate
Esmer et al.**^27^**	Systematic review	10 studies	Exercise programs (kinesiotherapy, aerobic exercise, resistance, and hydrotherapy).	Structured exercise improved muscle function, reduced pain, and enhanced QoL.	Level 1-2 – high
Annunziata et al.**^28^**	Expert consensus	Review and expert recommendations	International consensus document.	Regular physical activity benefited patients with lipedema by improving mitochondrial function and lymphatic circulation and by reducing systemic inflammation.	Level 6 – consensus/guideline
***Manual therapy***					
Herbst et al.**^29^**	Prospective pilot study, single arm	7 women with lipedema	12 90-min sessions of SAT therapy over 4 weeks.	Significantly reduced leg volume, skinfold thickness, pain, and tissue fibrosis; improved LEFS score and SAT structure.	Level 4 – low to moderate
Ibarra et al.**^30^**	Prospective pilot study, single arm	6 women with lipedema and 1 with Dercum’s disease	12 90-min sessions of SAT therapy over 4 weeks.	Significantly reduced leg fat mass (DXA), leg volume, and tissue edema; improved tissue structure and fascial reorganization (US); no significant change in pain (VAS).	Level 4 – low to moderate
***Shock wave therapy***					
Siems et al.**^31^**	Quasi-experimental study	26 women with lipedema and/or cellulite	Pneumatic ESWT combined with CDT, 6 sessions over 2 weeks.	Reduced oxidative stress (↓MDA, ↓carbonyls); improved skin elasticity and biomechanical properties.	Level 3 – moderate
Michelini et al.**^32^**	Observational pilot study	15 women with stage 2 lipedema (types II, III, and V)	8 sessions of ESWT (defocused + radial) combined with mesotherapy (Lymdiaral®), kinesio taping, and compression between sessions.	Reduced pain (NRS), circumference, and adipose tissue thickness; improved elasticity, US pattern, and QoL.	Level 4 – low to moderate
***Photobiomodulation***					
Baxter et al.**^33^**	RCT	17 women with breast cancer-related lymphedema	PBM (980/810 nm, 6 J/cm^2^, 12 sessions over 6 weeks) + conventional therapy vs. conventional therapy alone.	High adherence (>88%), no severe adverse events; no significant difference in limb volume or circumference, but a trend toward symptom and psychological improvement in the PBM group.	Level 2 – moderate
Chen et al.**^34^**	Systematic review and meta-analysis of RCTs	316 patients with post-mastectomy lymphedema (9 RCTs)	PBM (650/1000 nm, 1.5-6 J/cm^2^) vs. sham laser or conventional treatment.	Slight improvement in limb circumference and volume, not statistically significant; moderate to high heterogeneity; no severe adverse events.	Level 1 – high
Chiu et al.**^35^**	Systematic review and meta-analysis of RCTs	11 RCTs (breast cancer-related lymphedema)	PBM/LLLT (650/1000 nm, 1.5-6 J/cm^2^, 8-26 sessions, 2-3 ×/week) vs. sham or conventional therapy.	PBM reduced limb swelling and improved QoL; best results with axillary application, 3 sessions/week, 1.5-2 J/cm^2^, and >15 sessions; no severe adverse events.	Level 1 – high
Smoot et al.**^36^**	Systematic review and meta-analysis	8 RCTs (160 patients with lymphedema)	LLLT vs. placebo or MLD alone.	Significant improvement in limb volume, edema, pain, and function; greater effect when combined with compression.	Level 1 – high
***Intermittent pneumatic compression***					
Szolnoky et al.**^37^**	Quasi-experimental controlled pilot study	38 women with lipedema (19 intervention, 19 control)	CDT (MLD, compression, skin care, exercise) combined with IPC (30 min/day for 5 days).	Significant reduction in limb volume (≈ 5.6%) and pain intensity across all scales compared with control; no adverse events reported.	Level 3 – moderate
Wright et al.**^38^**	RCT	26 women with lipedema and secondary lymphedema	Pneumatic compression device + conservative care (compression stockings, MLD, education) vs. conservative care alone.	IPC promoted a 2-fold greater volume reduction compared with compression stockings alone.	Level 2 – moderate to high
Herbst et al.**^39^**	RCT	46 women with stage II lipedema	Home-based IPC group (30 days) vs. control group (compression stockings).	Significant reductions in left leg volume, total body water, extracellular and intracellular fluids, and subcutaneous tissue thickness; improvements in pain, edema, bruising, and QoL.	Level 1 – high
***Mechanical vibrotherapy***					
Schneider**^40^**	Randomized pragmatic clinical trial	30 women with lipedema	MLD alone vs. MLD combined with low-frequency vibrotherapy (15-42 Hz, 6 sessions, 30 min each).	Significant reduction in circumference in the ankle, calf, and thigh; improved QoL and feeling of lightness in the legs.	Level 1 – high

**Note:** Levels of evidence were classified according to the hierarchy proposed by Souza et al.,^13^ adapted for physical therapy research. **QuASiL**: Lipedema Symptom Assessment Questionnaire; **CDT**: complete decongestive therapy; **QoL**: quality of life; **VAS**: visual analog scale; **US**: ultrasound; **LEFS**: Lower Extremity Functional Scale; **6MWT**: Six-Minute Walk Test; **MRI**: magnetic resonance imaging; **PBM**: photobiomodulation; **RCT**: randomized controlled trial; **LLLT**: low-level laser therapy; **ESWT**: extracorporeal shock wave therapy; **MDA**: malondialdehyde; **NRS**: numerical rating scale; **MLD**: manual lymphatic drainage; **IPC**: intermittent pneumatic compression; **SAT**: subcutaneous adipose tissue; **DXA**: dual-energy X-ray absorptiometry; **SF-36**: 36-Item Short Form Health Survey; **PSFS**: Patient-Specific Functional Scale; **RAND-36**: RAND 36-Item Health Survey; **S2k**: German S2k Guideline.

## DISCUSSION

The findings of this integrative review were synthesized and discussed according to thematic categories, allowing comparison with the existing body of literature. This approach facilitated the identification of current evidence and knowledge gaps, as well as the characterization of the main physical therapy strategies used in the assessment and management of lipedema.

### Physical therapy assessment and diagnosis of lipedema

Given the clinical complexity and insidious progression of lipedema, physical therapy assessment becomes a fundamental step in outlining individualized and effective therapeutic approaches. In addition to contributing to differential diagnosis, the physical therapist is responsible for identifying functional and structural impairments, pain-related symptoms, and psychosocial repercussions, enabling a treatment plan tailored to the patient’s specific needs.^41,42^

The assessment process involves a detailed clinical history, including family history and age at symptom onset, which is often related to hormonal changes such as puberty, pregnancy, or menopause. Particular attention should also be given to symptoms commonly associated with lipedema, including pain, a feeling of heaviness, functional limitations, and reduced quality of life.^5,43^

Physical examination is an essential component of the diagnostic process and should be performed systematically. Characteristic findings include disproportionate enlargement of the lower extremities relative to the upper trunk, increased tenderness to touch, and pain upon palpation, even in the absence of acute inflammatory signs. Another common finding is spontaneous bruising or bruising after minimal trauma, attributed to the capillary fragility characteristic of lipedema.^3,6-8^ On examination, the Godet sign, characterized by the formation of a skin depression after digital pressure, is usually negative, indicating the absence of persistent indentation (pitting edema) in the compressed area, which helps distinguish lipedema from lymphatic or venous edema.^44^ Likewise, the Stemmer sign, assessed by attempting to pinch the skin at the base of the second toe or finger, is generally negative, demonstrating the absence of dorsal skin thickening and supporting the differential diagnosis from lymphedema.^3,6,45^

In addition to these findings, physical therapists should assess potential musculoskeletal and gait impairments resulting from chronic mechanical overload of the lower extremities. These evaluations are crucial to ensure an accurate physical therapy differential diagnosis, especially during the early stages of the disease, when clinical manifestations may resemble those of other edematous conditions.^5,10,15,45^

**Figure 2:**
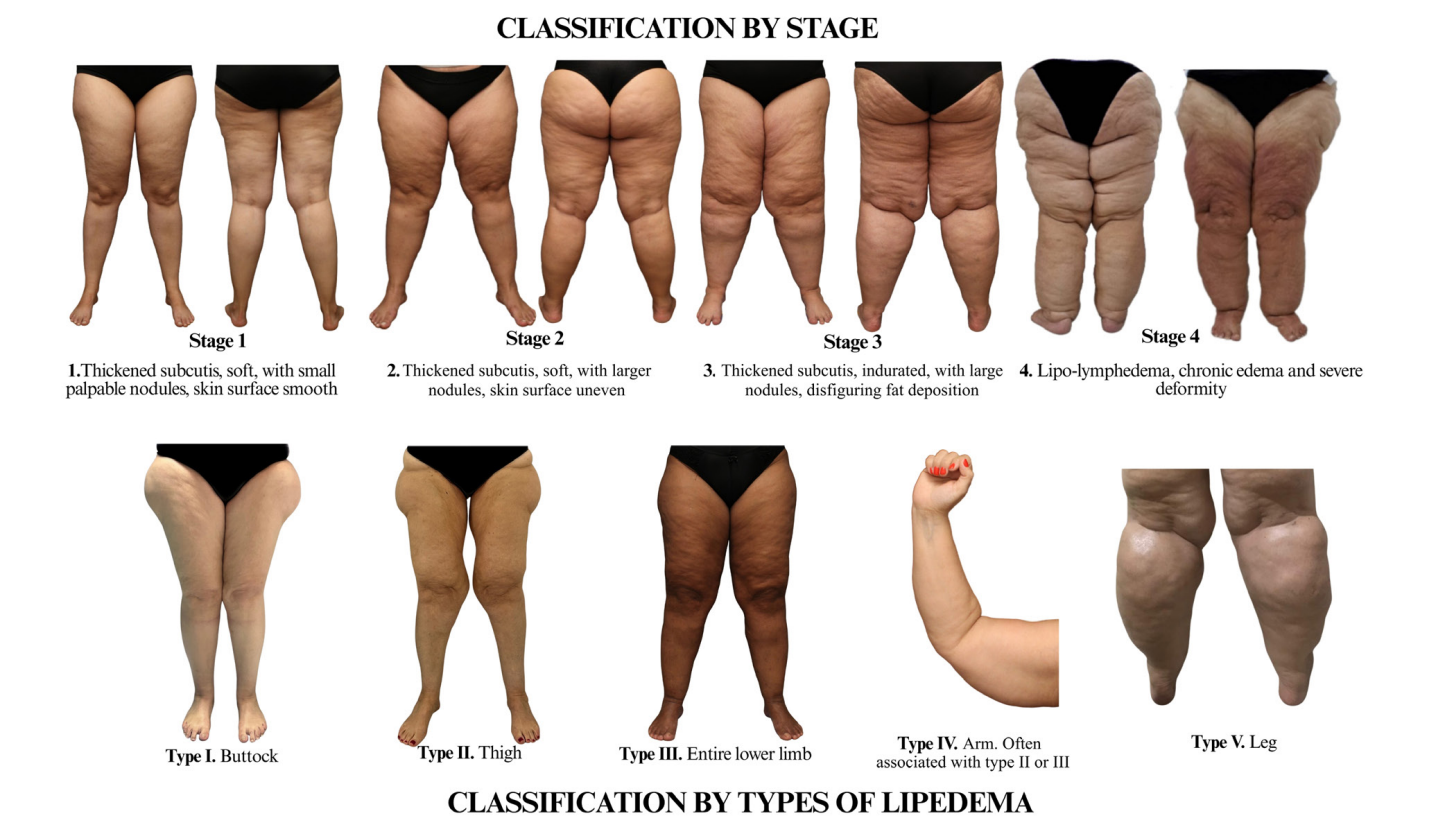
Clinical stages and anatomical types of lipedema according to the severity of subcutaneous tissue alterations and patterns of fat distribution. Images were obtained from patients who provided written informed consent for publication and use in scientific dissemination, in accordance with the ethical requirements of the journal. Source: Authors’ personal archive.

Based on clinical and functional findings, lipedema severity can be classified according to the appearance of the skin surface and structural changes in the adipose tissue of the extremities. In stage 1, the skin surface appears smooth despite a slight increase in subcutaneous adipose tissue. In stage 2, irregularities and palpable nodules are present throughout the affected areas. In stage 3, large extrusions of adipose tissue into the dermis and skin folds cause visible and more pronounced deformities (Figure 2).^10,11,44^

In addition to staging, Schingale^44^ proposed 5 anatomical types of lipedema. In type I, fat accumulation is limited to the buttocks and thighs. In type II, fat accumulation extends to the knees, with marked deposits in the medial region. In type III, involvement is continuous from the hips to the ankles. In type IV, both upper and lower extremities are affected. In type V, lipo-lymphedema occurs, characterized by the coexistence of lipedema and lymphedema (Figure 2).

Given the absence of specific laboratory biomarkers for lipedema, the use of standardized assessment instruments is essential to ensure a comprehensive and reproducible physical therapy evaluation. The combination of objective measurements and patient-reported outcome measures facilitates monitoring of disease progression, quantification of symptoms, and documentation of treatment responses, while providing comparative parameters that assist in clinical decision-making and in tailoring treatment strategies.

Among objective assessment methods, the estimation of segmental volume using the truncated cone formula, proposed by Kuhnke in 1976, remains one of the most widely used techniques. Originally developed for lymphedema assessment, this method has also been applied to lipedema because it enables objective quantification of limb volume, contributing to monitoring of disease progression and response to therapeutic interventions. The method assumes that limb segments resemble truncated cones and uses circumference measurements obtained at regular intervals, typically every 4 or 5 cm. It is a simple, noninvasive, and low-cost technique that provides objective and reproducible data, favoring individualized follow-up and optimization of physical therapy approaches.^10,45^ Additionally, standardized anthropometric measurements, such as thigh, leg, and ankle circumferences, also provide useful information for monitoring clinical outcomes and treatment responses over time.

Subjective pain intensity is commonly assessed using the visual analog scale (VAS), originally proposed by Huskisson in 1974. It consists of a continuous line anchored at each end by the descriptors “no pain” and “worst imaginable pain,” on which patients indicate their perceived pain intensity at the time of assessment.^10,15^

Among subjective lipedema-specific instruments, the Lipedema Symptom Assessment Questionnaire (QuASiL) stands out, as it was developed and validated to measure the intensity of disease-related symptoms and their impact on patients’ quality of life. The questionnaire consists of 15 items assessing manifestations such as pain, tenderness, bruising, heaviness, fatigue, and edema. Each item is scored on a scale ranging from 0 (no symptoms) to 10 (maximum symptom intensity), with a total score of up to 150 points. Higher scores indicate greater clinical and functional impairment.^14^

Another complementary instrument is the Western Ontario and McMaster Universities Osteoarthritis Index (WOMAC), originally developed by Bellamy et al. in 1988 for osteoarthritis assessment. Although not specific to lipedema, it has been used to evaluate lower-extremity disorders because it addresses aspects related to pain, joint stiffness, and physical function. The questionnaire contains 24 items divided into 3 domains (pain, stiffness, and functional capacity), each scored from 0 to 4. Domain scores can be summed up to generate a total score, where higher scores indicate greater functional impairment.^10,15^

Quality of life is frequently assessed using the 36-Item Short Form Health Survey (SF-36), developed by Ware and Sherbourne in 1992. The instrument includes 8 health domains, and the resulting scores generate 2 summary components (physical and mental health) that provide a comprehensive view of patient-perceived health status.^10,15^

Visual documentation can be produced by obtaining standardized serial clinical photographs under consistent positioning and lighting conditions, allowing for objective comparisons over time. Additional assessment tools include segmental bioelectrical impedance analysis, which estimates body composition and fluid accumulation, and bioimpedance vector analysis, which provides resistance and reactance measurements for the calculation of phase angle and assessment of cellular integrity. Soft-tissue ultrasound can further contribute to the characterization of tissue density, organization, and integrity, being particularly valuable in specialized clinical settings.^10,15^

Accurate physical therapy assessment and diagnosis also require differentiation of lipedema from other disorders with overlapping clinical features. The main differential diagnoses include lymphedema, typically characterized by asymmetric edema, positive Godet sign, foot involvement, and trophic skin changes; obesity, which involves diffuse and generally painless fat accumulation without spontaneous bruising; chronic venous insufficiency, which manifests as orthostatic edema, varicose veins, and skin changes such as hyperpigmentation and lipodermatosclerosis; lipohypertrophy, involving localized increases in subcutaneous adipose tissue without pain, bruising, or vascular compromise; and systemic edema of cardiac, renal, or hepatic origin, which should be excluded based on clinical history, complementary examinations, and assessment of associated systemic manifestations.^10,14-16,45^

Correct identification of lipedema is essential for appropriate treatment planning and for establishing individualized physical therapy goals.

#### Evidence summary

Most assessment methods used in lipedema currently rely on Level 4 evidence (low to moderate). The QuASiL questionnaire shows comparatively stronger methodological support (Level 1-2). Ultrasound and bioimpedance techniques show preliminary to moderate evidence for tissue monitoring, whereas clinical staging systems, anatomical classifications, and differential diagnostic signs continue to be supported primarily by descriptive studies and expert consensus.

### Physical therapy interventions

Physical therapy plays a key role in the conservative treatment of lipedema through the use of interventions such as manual therapies, therapeutic exercise, and electrophysical agents to promote homeostasis of the affected systems. Given the multifactorial nature of the disease, which involves changes in adipose tissue structure and function, lymphatic and venous microcirculatory dysfunction, and a chronic low-grade inflammatory state, these noninvasive, evidence-based interventions can address multiple components of the clinical presentation simultaneously.

Treatment planning should begin with a comprehensive assessment that considers disease stage, functional status, and associated comorbidities. Based on this evaluation, individualized treatment goals can be established, including reduction in limb volume, pain relief, improvement of mobility and muscle strength, preservation of skin integrity, prevention of complications and disease progression, and enhancement of patients’ functional capacity and quality of life.

Currently, the most widely adopted physical therapy approach is based on complete decongestive therapy (CDT), which is considered the gold standard conservative treatment for lymphedema. However, because important pathophysiological differences exist between lymphedema and lipedema, adaptations to the traditional CDT model are necessary to address the specificities of lipedema.^14-16^

### Complete decongestive therapy

CDT, also referred to as complex decongestive physiotherapy (CDP), is widely recognized as a first-line conservative approach for advanced lipedema and lipo-lymphedema.^15,27^ Although lipedema is not primarily classified as a lymphatic disease, evidence of microcirculatory impairment and superficial lymphatic capillary dilation provides a rationale for the use of this approach.^27^ International societies, such as the International Lipoedema Association,^20^ as well as authors such as Schmeller and Meier-Vollrath,^21^ recommend CDT as an initial intervention, with the possibility of combining it with adjunctive strategies such as intermittent pneumatic compression (IPC) and psychological support.

CDT is based on 4 therapeutic components: manual lymphatic drainage (MLD), compression therapy with multilayer bandaging or elastic stockings, myolymphokinetic exercises, and skin care. In lipedema, these components should be adapted to the clinical characteristics of the disease, such as pain, hypersensitivity, and adipose tissue changes. Compression therapy, in particular, plays a central role in tissue containment, pain relief, and reduction in the risk of progression to lipo-lymphedema in advanced stages, provided that it is appropriately prescribed and monitored.^20,21^

Clinical studies support the potential benefits of CDT in lipedema. Szolnoky et al.^17^ evaluated 21 women who underwent a 5-day protocol including MLD, IPC, skin care, and multilayer bandaging, and reported reductions in limb volume and capillary fragility. In a randomized controlled trial involving 33 women, Atan and Bahar-Özdemir^18^ showed that CDT combined with exercise was significantly more effective in reducing limb volume and pain and improving physical function than exercise or IPC alone. Similar findings were reported by Donahue et al.^19^ in a pilot study of 5 women with early-stage lipedema who completed 9 sessions of conventional physical therapy, including CDT, individualized exercise, education, and compression. The intervention was associated with pain relief, functional improvement, and reduced sodium deposition in adipose tissue, suggesting a decrease in local inflammation.

Supervised physical exercises, such as walking, low-intensity cycling, and hydrotherapy, are recommended as part of the CDT protocol because they may enhance venous and lymphatic return and reduce subcutaneous tissue stiffness. Preventive skin care is also essential, especially in advanced stages, to reduce the risk of secondary infections. International guidelines, including the American guideline, further support the applicability of CDT and highlight its positive effects on lymphatic drainage, edema control, pain relief, and tissue preservation.^22^

#### Evidence summary

The available evidence supporting CDT for lipedema is Level 3-4 (moderate to low). Nevertheless, international consensus statements and clinical guidelines (Level 6) recognize CDT as the main conservative approach, with potential benefits in reducing pain and limb volume, improving physical function, and contributing to disease progression control when applied as part of an integrated therapeutic strategy.

### Compression therapy

Compression therapy is considered one of the main conservative strategies in the management of lipedema, alongside MLD, therapeutic exercise, and functional re-education. Although lipedema is not classically defined as an edematous disease in its early stages, compression has proven effective in controlling pain, improving mobility, preventing fibrosis, and promoting lymphatic and venous return particularly in patients with signs of lymphatic involvement or advanced disease.^18,44^ Compression therapy also plays an essential role in clinical management because signs of lymphatic dysfunction, such as the dilatation of lymphatic collectors and dermal backflow, may be present even in the early stages of lipedema.^5^

Rasmussen et al.^46^ evaluated lymphatic function in 20 women with stage 1 and 2 lipedema using near-infrared fluorescence lymphatic imaging and identified dilated lymphatic vessels and intravascular pooling, highlighting the importance of external compression from the early stages of the disease. Similarly, Chachaj et al.^47^ performed lymphoscintigraphy in 51 women with lipedema and 31 women with obesity and demonstrated that, although both groups exhibited evidence of lymphatic overload, the abnormalities were more pronounced in patients with lipedema.

The relationship between lymphatic dysfunction and fibrosis has also been extensively investigated. Avraham et al.^48^ proposed that impaired lymphatic drainage promotes collagen deposition in the extracellular matrix, thus worsening the fibrotic condition. Brorson^49^ demonstrated that compression therapy may help maintain the benefits achieved through volume reduction treatments while preserving lymphatic function. Therefore, controlling interstitial edema is fundamental, as lymphatic stasis promotes chronic inflammation, fibrosis deposition, and adipocyte hyperplasia.

Based on the available evidence, clinical guidelines have proposed compression protocols according to disease severity. For stage 1 lipedema, compression garments providing 10 to 20 mm Hg are indicated; for stages 2 and 3, compression levels between 20 and 40 mm Hg are preferred; and for stage 4 (lipo-lymphedema), multilayer bandaging is recommended. In contrast, patients with early-stage lipedema and no clinically significant edema typically benefit from lower compression levels, with emphasis placed on symptom management and patient tolerance.^5^

Czerwińska et al.^23^ further emphasized that both the type of garment (flat-knit, circular-knit, or micromassage compression) and the level of compression should be individualized according to clinical stage, limb morphology, symptom severity, and patient tolerance.

In stage 4 disease, multilayer bandaging is the primary compression strategy. This approach uses short-stretch bandages to generate high working pressures during movement and should be administered by properly trained physical therapists. Continuous clinical monitoring is essential to allow treatment adjustments according to patient progress. Furthermore, combining compression therapy with physical exercise and lymphatic drainage may enhance therapeutic outcomes. This approach requires appropriate clinical indication, individualized fitting, and continuous monitoring of treatment effects.^5,15,18^

In addition to its well-established physiological effects, compression therapy may also influence the neurosensory system. Studies have demonstrated that the pressure exerted on the skin, muscles, and joints generates sensory input that is processed by the central nervous system, contributing to improved body support, postural stability, pain modulation, and mobility.^24,25^ In a survey involving 279 women with lipedema, Paling and Macintyre^24^ reported perceived benefits including reductions in pain and swelling; however, participants also identified challenges such as discomfort and poor garment fit due to their limb morphology.

Similarly, Ricolfi et al.^25^ reported that the use of micromassage compression leggings combined with physical exercise was associated with reduced limb volume, improved pain, and enhanced skin texture. Despite these benefits, treatment adherence may still be limited by factors such as hyperalgesia and difficulty in donning compression garments. Balcombe et al.^50^ suggested strategies to address these barriers, including adjustable Velcro compression systems, donning aids, and functional re-education.

Physical therapists play a central role in the prescription and management of compression therapy by providing patient education, adapting interventions to individual needs, and monitoring therapeutic outcomes. Effective implementation requires a thorough understanding of the indications, garment types, and compression levels available, ensuring individualized and clinically appropriate treatment according to disease stage and patient-specific characteristics.

#### Evidence summary

The available evidence supporting compression therapy in lipedema is predominantly classified as Level 3-4 (moderate to low). Nevertheless, international consensus statements and clinical guidelines (Level 6) recognize compression therapy as an essential component of conservative management. The greatest benefits appear to be achieved when compression is individualized according to disease stage, with higher compression levels reserved for advanced lipedema and lipo-lymphedema.

### Motor and musculoskeletal physical therapy

Motor and musculoskeletal physical therapy represents an important component of lipedema management, addressing not only the abnormal accumulation of subcutaneous adipose tissue but also the biomechanical and functional repercussions of the disease. Individuals with lipedema often present with chronic pain, muscle weakness, and joint dysfunction, all of which compromise mobility and quality of life.

van Esch-Smeenge et al.^26^ demonstrated that women with lipedema exhibit significantly lower quadriceps muscle strength than women with obesity, indicating that muscle weakness and reduced endurance may contribute directly to impaired functional capacity. Similarly, the review by Esmer et al.^27^ reported that structured exercise programs, including kinesiotherapy, resistance training, aerobic exercise, hydrotherapy, and postural re-education, are effective in improving muscle function, reducing pain, and enhancing quality of life.

Beyond mechanical benefits, physical exercise also modulates chronic inflammation associated with lipedema. Exercise-induced increases in interleukin (IL)-6 have been shown to reduce tumor necrosis factor-alpha (TNF-α) production while stimulating the release of anti-inflammatory cytokines, including IL-10 and IL-1 receptor antagonist (IL-1ra). This physiological mechanism helps control persistent inflammation, regulates adipose tissue metabolism, and promotes positive functional adaptations.^26,27^

These findings are supported by the joint consensus of the Italian Society of Motor and Sports Sciences (SISMeS) and the Italian Society of Phlebology (SIF),^28^ which emphasizes that, although lipedema has historically been considered resistant to exercise-based interventions, regular physical activity can optimize muscle function, improve lymphatic circulation, modulate systemic inflammation, and prevent osteoarticular complications.

Therefore, motor and musculoskeletal physical therapy should prioritize interventions aimed at muscle strengthening, supervised aerobic conditioning, and functional re-education. When appropriately prescribed and adapted to individual needs, these strategies may improve physical performance, preserve mobility, and contribute positively to long-term disease management.

#### Evidence summary

The available evidence supporting motor and musculoskeletal physical therapy in lipedema is predominantly classified as Level 3-4 (moderate to low). Nonetheless, international consensus statements and clinical guidelines (Level 6) consistently highlight structured physical exercise as a key component of conservative management, particularly when interventions are individualized according to disease stage and functional status.

### Manual therapy

Tissue fibrosis, which may be present as early as stage 1 lipedema, poses a significant challenge in disease management. Interstitial fibrosis and extracellular matrix deposition can impair lymphatic drainage and reduce therapeutic effectiveness.^51-53^

In this context, manual therapy has emerged as a valuable therapeutic approach aimed at promoting extracellular matrix remodeling, improving interstitial fluid circulation, and reducing adipose tissue stiffness. Several techniques have been described in the literature, including MLD, myofascial release, mobilization of subcutaneous adipose tissue (SAT therapy), and negative pressure therapy, all with potential to improve tissue mobility and lymphatic flow.^51-53^

Herbst et al.^29^ investigated the effects of SAT therapy in 7 women with lipedema using connective tissue remodeling and drainage-oriented techniques over a 4-week period. The intervention resulted in reductions in limb and trunk volume, fibrosis, and pain sensitivity, indicating tissue restructuring and symptom relief.

Similarly, Ibarra et al.^30^ evaluated SAT therapy in 7 women with lipedema and 1 woman with Dercum’s disease, who underwent 12 90-minute treatment sessions over 4 weeks. Ultrasound and dual-energy X-ray absorptiometry (DXA) scans showed reductions in leg weight, total leg volume, and tissue stiffness, along with improvements in subcutaneous tissue structure and mobility.

Manual therapy appears to contribute to collagen remodeling and reduced extracellular protein deposition, minimizing the progression of fibrosis. When integrated with other physical therapy strategies, such as therapeutic exercise and CDT, these approaches may optimize clinical outcomes in individuals with lipedema.

#### Evidence summary

The current evidence supporting manual therapy in lipedema is Level 4 (low to moderate). Pilot studies indicate that SAT therapy may reduce limb volume, fibrosis, and pain sensitivity while improving tissue structure and mobility. Although the available evidence is limited by small sample sizes, the findings support manual therapy as a valuable complementary approach in conservative lipedema management.

### Electrophysical agents

Physical therapy offers a variety of electrophysical agents that generate electrical, mechanical, thermal, and non-thermal stimuli, producing therapeutic effects that can modulate physiological processes, stimulate tissue repair, and influence adipose tissue metabolism. However, their use in the treatment of lipedema requires caution because of the specific pathophysiological characteristics of this condition. Although these modalities are widely used for tissue remodeling and edema reduction in other disorders, evidence confirming their safety and efficacy in lipedema is still limited. Therefore, the selection of electrophysical modalities should consider not only their potential benefits but also the risks associated with inappropriate stimulation, which may exacerbate the inflammatory process, adipocyte dysfunction, and capillary fragility characteristic of lipedema.

### Shock wave therapy

Among the most studied modalities in the context of lipedema, extracorporeal shock wave therapy (ESWT) has received particular attention due to its anti-inflammatory, tissue remodeling, and antifibrotic potential. Previous studies have demonstrated the positive effects of ESWT in lymphedema, including fibrosis reduction and induction of lymphangiogenesis, supporting its investigation as a potential therapeutic approach for lipedema. Furthermore, ESWT appears to influence adipose tissue metabolism by promoting autophagic lipolysis via apoptosis, which may contribute to tissue remodeling and reduction of dysfunctional fat accumulation.^31,32^

Siems et al.^31^ evaluated the effects of pneumatic ESWT combined with CDT in 26 patients with lipedema and cellulite. The protocol consisted of 2 sessions per week for 2 weeks, using an energy flux density of 0.16 mJ/mm^2^, a 30 mm applicator, and 1000 pulses per session applied to the thighs. CDT was administered daily to both thighs, whereas ESWT was applied unilaterally for comparison. The study showed that ESWT reduced oxidative stress, as indicated by reduced lipid peroxidation and decreased malondialdehyde (MDA) levels, a biomarker of oxidative stress and cellular damage. In addition, a reduction in fibrogenic cytokines suggested an antifibrotic effect. Another relevant finding was the improvement in the biomechanical properties of the skin, including increased elasticity and reduced interstitial fibrosis, factors that may facilitate lymphatic drainage and improve tissue function.

Michelini et al.^32^ investigated ESWT combined with mesotherapy and kinesio taping in a pilot study of 15 women diagnosed with stage 2 lipedema (types II, III, and V). The protocol consisted of 8 sessions held twice weekly, combining defocused and radial electromagnetic shock waves (3400 and 5000 pulses, respectively), intradermal injections of Lymdiaral®, a homeopathic herbal compound, and kinesio taping applied from proximal to distal regions, covering the trochanteric region, posterior thighs, and calves. The tape was maintained for 3 days and reapplied at the subsequent session. In addition, 20–25 mm Hg compression stockings were recommended between sessions to optimize lymphatic drainage. The results showed significant reductions in pain, subcutaneous adipose tissue thickness, and circumference measurements, as well as improved tissue elasticity, reflecting a positive impact on patients’ quality of life.

The beneficial findings reported in clinical studies on ESWT in lipedema are consistent with mechanistic research investigating its effects on adipose tissue, skin, and inflammatory profiles. These studies suggest that ESWT stimulates adipocyte metabolism, reduces inflammation, and supports tissue repair.^54,55^ Together, these effects indicate that ESWT may be a promising therapeutic tool in the management of lipedema, especially in patients with fibrosis associated with lymphatic dysfunction.

#### Evidence summary

The current evidence supporting ESWT in lipedema is predominantly Level 3-4 (moderate to low). Small clinical studies indicate potential benefits in reducing pain, subcutaneous adipose tissue thickness, limb circumference, and fibrosis, as well as improving skin elasticity. Although the available evidence remains limited, current findings suggest that ESWT may represent a promising adjunctive strategy when combined with other conservative interventions.

### Photobiomodulation

Photobiomodulation (PBM) has been widely investigated in the management of lymphedema due to its ability to modulate inflammation, reduce limb volume, relieve pain, and minimize tissue fibrosis. Studies conducted by Baxter et al.,^33^ Chen et al.,^34^ and Chiu et al.^35^ have demonstrated that combining PBM with compression-based physical therapy significantly enhances clinical outcomes. Given its established benefits in lymphedema, there is biological plausibility for its application in the treatment of lipedema.

In addition to its recognized clinical effects, recent evidence suggests that PBM acts on the mitochondrial pathway, triggering a cascade of cellular events that promote increased metabolism of superficial adipose tissue. The systematic review and meta-analysis conducted by Smoot et al.^36^ confirmed that low-level laser PBM can improve lymphatic drainage, reduce edema and pain, and positively impact the function of the affected limb.

These mechanisms provide further support for the potential application of PBM in lipedema. By enhancing lymphatic drainage and promoting tissue regeneration, PBM may target key pathophysiological features of the disease, such as adipocyte hypertrophy, interstitial fibrosis, and chronic inflammation.^34-36^ As a non-thermal modality, PBM represents a promising option in the conservative management of lipedema. However, further studies are still needed to define optimal treatment parameters and to establish its clinical efficacy specifically in individuals with lipedema.

#### Evidence summary

The evidence supporting the use of PBM in lipedema is currently indirect and derives from clinical studies conducted in patients with lymphedema, with evidence levels ranging from 1 to 2 (high to moderate). These studies suggest potential benefits in reducing pain, edema, and tissue fibrosis, while improving lymphatic drainage and overall tissue quality. Although lipedema-specific evidence remains limited, the available findings suggest that PBM may represent a consistent, safe, and potentially valuable approach in the conservative management of lipedema.

### Intermittent pneumatic compression

IPC is a therapeutic modality employed in the management of venous and lymphatic disorders. In the context of lipedema, IPC has gained prominence as part of a multimodal approach aimed at reducing symptoms, enhancing lymphatic drainage, relieving pain, and controlling capillary fragility, which contributes to the frequent occurrence of bruising in affected individuals.^19,46^

IPC involves the external application of sequential and graduated pressures using a pneumatic pump connected to inflatable sleeves that are wrapped around the affected limbs. Cycles of inflation and deflation mimic physiological pumping, thus facilitating venous and lymphatic return and promoting mechanical mobilization of interstitial fluid.^38,39^

Although lipedema does not present with classic lymphatic edema in its early stages, evidence suggests the presence of secondary lymphatic dysfunction in more advanced stages, in addition to inflammatory and microvascular changes that contribute to pain and functional impairment.^19,46^ In this context, IPC serves as an adjuvant therapeutic resource by improving circulation and reducing tissue overload.

Szolnoky et al.^37^ conducted a comparative study to evaluate the efficacy of IPC versus CDT in the treatment of lipedema. Twenty-three women were allocated to 1 of 2 treatment groups: one received CDT alone (60 minutes daily), whereas the other received a combined protocol consisting of 30 minutes of CDT followed by 30 minutes of IPC. Both interventions were effective in reducing limb volume and improving capillary fragility, providing symptomatic relief. Although promising, this was a pilot study with a small sample size, indicating the need for further research with stronger methodological rigor.

Additionally, Wright et al.^38^ investigated the effects of pressotherapy in lipedema management and observed that patients treated with IPC achieved twice the reduction in limb volume compared with those using compression stockings alone. These findings support IPC as a promising therapeutic strategy in the conservative treatment of lipedema.

More recently, Herbst et al.^39^ conducted a randomized controlled trial to isolate the effects of IPC using an advanced pneumatic compression device in women with stage 2 lipedema without clinically evident lymphedema. The study included 46 participants who were assigned either to an intervention group, which used the device at home for 30 days, or to a control group, which maintained only the use of compression stockings. The intervention group showed significant reductions in left leg volume, total body water, extracellular and intracellular fluid volumes, and subcutaneous adipose tissue depth in various regions as assessed by ultrasound. Clinically meaningful improvements were also observed in pain, swelling, bruising, quality of life, and functional status compared with the control group. These findings support the effectiveness of IPC as a relevant therapeutic strategy in lipedema management, especially when combined with continuous elastic compression.

Despite these promising results, IPC should not be considered a replacement for MLD or conventional compression therapy. Rather, it should be indicated as a complementary intervention within an integrated care plan. Its use should be guided by the clinical stage of lipedema, symptom severity, and the patient’s tolerance and ability to adhere to treatment.

#### Evidence summary

The available evidence supporting IPC in lipedema is Level 1-3 (moderate to high). Randomized and comparative clinical studies have reported reductions in limb volume, edema, and pain, as well as improvements in quality of life, especially when combined with elastic compression or CDT. Although further studies are needed to standardize treatment parameters and confirm long-term outcomes, current evidence suggests that IPC is a safe intervention in the conservative management of lipedema.

### Mechanical vibrotherapy

Vibrotherapy has been investigated as an adjuvant resource in the physical therapy management of lipedema, particularly due to its potential effects on lymphatic drainage, interstitial pressure reduction, and quality-of-life improvement. Vibratory devices used in this approach may be applied locally or delivered through adjustable whole-body platforms that generate low-frequency vibrational impulses throughout the body.

Schneider^40^ evaluated the effects of whole-body mechanical vibrotherapy in 30 women diagnosed with stage 2 and 3 lipedema. The intervention was performed using an adjustable vibration table, with frequencies ranging from 15 to 42 Hz, in combination with MLD. Participants were randomized into 2 groups: one received MLD alone, whereas the other received MLD combined with vibrotherapy. The results showed that the addition of vibrotherapy significantly enhanced the effects of MLD, with reductions in circumference ranging from 1.1 to 3.2 cm in the ankles, calves, and thighs compared with the control group. Furthermore, patients who received the combined intervention reported improved quality of life and a greater feeling of lightness in their legs.

According to the author, the proposed physiological mechanisms underlying the benefits of vibrotherapy include reduction in interstitial pressure, traction of myofilaments, and opening of lymphatic capillaries, resulting in optimized lymphatic flow and mobilization of interstitial fluid. Although the results are promising, the study is considered preliminary, and further clinical trials are required to standardize treatment protocols, assess long-term efficacy, and elucidate the mechanisms involved in the use of vibrotherapy for the treatment of lipedema.

#### Evidence summary

The available evidence supporting mechanical vibrotherapy in lipedema is limited and derives from a single randomized controlled trial, classified as Level 1. This study suggested significant reductions in limb circumference and potential improvements in quality of life when vibrotherapy was combined with lymphatic drainage. Although these findings are promising, further studies are needed to standardize vibration parameters and confirm long-term effects in the management of lipedema.

## CONCLUSION

The findings of this integrative review provide an overview of the current landscape of evidence on the role of physical therapy in the management of lipedema. While the available literature remains limited and is predominantly composed of studies with moderate to low levels of evidence, the existing findings provide valuable guidance for clinical practice and contribute to the development of safer and more evidence-based therapeutic approaches. Nevertheless, further well-designed studies are needed to confirm these results and to strengthen the evidence base supporting the standardization of physical therapy approaches in the treatment of lipedema.
